# How AI Is Transforming Medical Education: Bibliometric Analysis

**DOI:** 10.2196/75911

**Published:** 2025-11-18

**Authors:** Youyang Wang, Chuheng Chang, Wen Shi, Huiting Liu, Xiaoming Huang, Yang Jiao

**Affiliations:** 1 Department of General Practice (General Internal Medicine) Peking Union Medical College Hospital, Chinese Academy of Medical Sciences & Peking Union Medical College Beijing China; 2 Department of Radiation Oncology National Cancer Center/National Clinical Research Center for Cancer/Cancer Hospital, Chinese Academy of Medical Sciences and Peking Union Medical College Beijing China; 3 Department of Gastroenterology Peking Union Medical College Hospital, Chinese Academy of Medical Sciences & Peking Union Medical College Beijing China; 4 Department of Infectious Diseases Peking Union Medical College Hospital, Chinese Academy of Medical Sciences & Peking Union Medical College Beijing China

**Keywords:** artificial intelligence, AI, medical education, bibliometric analysis, research trends, generative artificial intelligence, generative AI

## Abstract

**Background:**

Artificial intelligence (AI) is increasingly being integrated into medical education. As AI technologies continue to evolve, they are expected to enable more sophisticated student tutoring, performance evaluation, and reforms of curricula. However, medical education entities have been ill-prepared to embrace this technological revolution, and there is anxiety concerning its potential harm to the community.

**Objective:**

To explore research trends in the field and identify future directions for AI-enabled medical education, we conducted a systematic bibliometric analysis focusing on temporal trajectories in the field.

**Methods:**

Documents were collected from the Web of Science and Scopus databases covering the period from 2000 to 2024. A multistep search strategy combining information retrieval, a definitive journal list, and cocitation analysis was used to identify relevant publications. Journal and author impact were assessed using both publication and citation metrics. Research trends and hot spots were examined through citation burst detection, frequency analysis, and co-occurrence networks, with a color gradient used to indicate the average occurrence year of keywords. The citation lineage structure of the field was evaluated using a k-means clustering-based analysis of cocitation networks to trace influential references.

**Results:**

Our analysis revealed a significant increase in publications since 2021, with foundational works emerging as early as 2019. Influential journals in this domain included *JMIR Medical Education*, *Anatomical Sciences Education*, and *Medical Education*. The evolving research trajectory exhibited a shift from conventional computer-assisted learning tools toward generative AI platforms. Earlier applications of AI in medical education were predominantly concentrated at the undergraduate level, indicating substantial potential for expansion into graduate and continuing medical education. Furthermore, limited cocitation connections were observed between recent generative AI research and conventional medical AI studies, and investigations into medical students’ attitudes toward generative AI remain scarce.

**Conclusions:**

There are critical needs for (1) interdisciplinary studies that intentionally integrate generative AI with foundational medical AI work and (2) involving medical educators and students in AI development. Future research should focus on building theoretical frameworks and collaborative projects that connect these currently separate domains to foster a more cohesive knowledge base.

## Introduction

The integration of artificial intelligence (AI) into medical education is not just a passing trend but a transformative leap that promises to revolutionize the quality, efficiency, and accuracy of medical learning and practice. This integration not only addresses the challenges posed by the sheer volume and complexity of medical knowledge but can also offer personalized learning experiences tailored to individual students’ needs and abilities [1]. Nevertheless, there is still concern about AI’s role in the future of medical education. Ethical issues, including student privacy; bias inherent in the data or algorithms; and model explainability, transparency, and accountability, are legitimate concerns [2]. The possibility of being replaced by AI or losing control over machines also generates distrust among stakeholders in medical education [3]. While advanced, AI tools often focus on the technical aspects of education delivery, neglecting the nuanced intricacies of human cognition and learning [4].

While the scholarly conversation on this topic is growing rapidly, the existing literature lacks a comprehensive, data-driven map of its intellectual structure and evolution. Narrative reviews and bibliometric studies in adjacent fields such as general AI in health care or digital education tools exist [5,6], but a dedicated analysis that tracks the evolution of research hubs within this specific domain is missing. Key questions remain unanswered: What are the foundational papers that have shaped this field? How have research themes evolved over time? What are the current dominant clusters of knowledge, and where is the frontier heading?

This study conducted a systematic bibliometric analysis to delineate the structural and temporal dynamics of medical education technology research. Adopting the methodological framework by Maggio et al [7], we systematically analyzed journal and author impact through publication and citation metrics; identified research trends and hot spots using citation burst detection, keyword frequency analysis, and co-occurrence networks supplemented by average occurrence year; and traced influential reference lineages via k-means clustering of cocitation networks. The objective was to provide an evidence-based map of the field’s evolution, highlighting key areas of impact and emerging thematic directions to inform strategic research and educational planning.

## Methods

**Figure 1:**
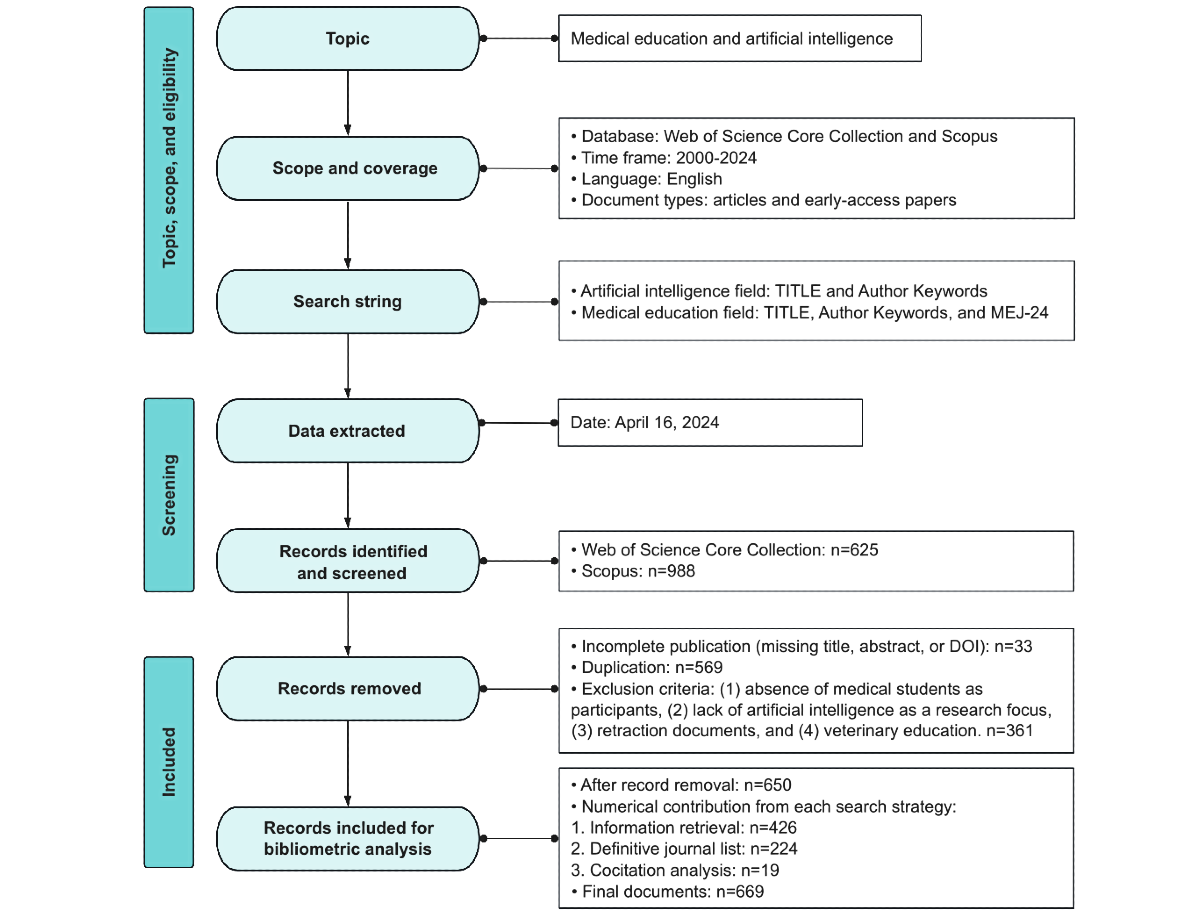
Study flowchart. *The Medical Education Journal List 24 (MEJ-24) was proposed by Maggio et al [7] and is provided in Multimedia Appendix 1.

This was a systematic bibliometric analysis, and the study selection process is illustrated in Figure 1 [7], which shows the identification, screening, and inclusion and exclusion criteria of documents.

### Ethical Considerations

This study did not involve human or animal participants and did not require patient consent. The ethics committee of Peking Union Medical College Hospital waived the ethical application because only published data were used. Patients or the public were not involved in the design, conduct, reporting, or dissemination plans of our research.

### Data Extraction

Documents were extracted from the Web of Science and Scopus databases. To refine the strategy, we used 3 complementary approaches to delineate the field of AI in medical education following the framework established by Maggio et al [7].

The first approach was information retrieval. Initially, search terms were identified by reviewing existing systematic reviews and key publications in the fields of AI and medical education [7,8]. This process established a baseline set of keywords, including “artificial intelligence,” “machine learning,” and “medical education.” The preliminary search string was then evaluated by a medical librarian and domain experts to ensure its appropriateness and was refined based on their feedback. This refinement involved incorporating more technical terms (eg, “deep learning,” “natural language processing,” and “transformer models”) to better capture emerging trends. Furthermore, Boolean operators and truncation (eg, “educat* OR curricul* OR teach*”) were applied to enhance search breadth. The revised search string was deployed using field searching techniques, specifically within the title and author keyword fields. The detailed search strategy is provided in Multimedia Appendix 2.

The second approach was definitive journals. Core medical education journals were identified using the Medical Education Journal List 24 (MEJ-24), an empirically derived set of journals validated through collaboration between medical education researchers and bibliometric experts [7]. For the search strategy, we conducted field searches across the 24 journals of the MEJ-24 using the journal source field. For AI-related terms, we searched the author keyword and title fields following the same information retrieval approach detailed previously. The MEJ-24 journals can be found in Multimedia Appendix 1.

The third approach was journal cocitation analysis. The journal cocitation analysis was conducted using the document pool retrieved through the 2 search strategies described previously (ie, information retrieval and the definitive journal list). Following data selection (as detailed in the Data Selection section), 650 documents were identified. A reference co-occurrence matrix was then constructed (see the Bibliometric Analysis section for details), and the top 30 most cited references among these articles were quantified. Notably, 63% (19/30) of these highly cited references had not been captured by the original search string. These 19 articles were subsequently added to the cohort.

Documents were collected from January 1, 2000, to April 16, 2024. Only articles in English, reviews, and early-access papers were included.

### Data Selection

The extraction approach yielded 1613 documents (n=625, 38.75% from Web of Science and n=988, 61.25% from Scopus). Documents lacking titles, abstracts, or digital object identifiers (DOIs) were removed, as were duplicates identified across the document pools using DOI matching. The inclusion criteria required that documents focus on the application of AI or computer-assisted techniques within medical education and present educational interventions or evaluations of AI tools in medical training contexts. Exclusions comprised retracted publications and documents addressing specific off-topic technical or noneducational themes. These included machine learning methods with nomenclature overlaps but no substantive educational relevance (eg, “teacher-student curriculum learning algorithm”), medically focused topics unrelated to AI in education (eg, “computer vision syndrome”), general online or remote education literature without specific AI components, and publications related to veterinary education. Our literature screening and selection process was conducted by 2 independent reviewers. Interrater reliability was quantified using the Cohen κ, which yielded a value of 0.85 based on 38.56% (622/1613) agreed inclusions, indicating strong agreement between reviewers. Discrepancies in inclusion decisions were resolved through consultation with 2 domain experts. Of the 107 articles over which there was initial disagreement, 28 (26.2%) were selected for inclusion after expert review, resulting in a total of 650 articles. A subsequent cocitation analysis of these 650 articles identified the 30 most cited references, 19 (63%) of which were not captured in the original search. These 19 articles were added to the cohort, bringing the final total to 669 articles (Multimedia Appendix 3). Information retrieval from databases contributed 63.7% (426/669) of the articles, the definitive journal list method contributed 33.5% (224/669) of the articles, and cocitation analysis contributed 2.8% (19/669) of the articles.

### Data Processing

We imported raw export files in CSV format from both Scopus and Web of Science into the R environment (version 4.4.2; R Foundation for Statistical Computing) and standardized column names across datasets to ensure compatibility. Formatting inconsistencies in titles, abstracts, keywords, and author lists were resolved to create consistent records across both databases. Following the data selection process (as described in the Data Selection section), the preprocessed dataset was manually screened by 2 independent reviewers using Microsoft Excel. Discrepancies regarding inclusion were resolved through consultation with domain experts.

During keyword normalization, terms were standardized by converting them to lower case, reconciling plural and singular forms, expanding abbreviations, and removing special characters. For example, variants such as “3d computer-models,” “3d models,” and “3d reconstruction” were merged into the unified term “3d model.” Keywords such as “artificial intelligence,” “medical students,” “computer,” and “technology” were removed due to their limited analytical value. A complete mapping of these keyword transformations is provided in Multimedia Appendix 4.

Finally, we constructed a comprehensive analysis-ready dataset containing standardized fields for DOI, title, abstract, keywords, citation counts, authors, journals, publication year, and references. This final matrix formed the basis for all subsequent bibliometric analyses.

### Bibliometric Analysis

Quantitative assessments of authors, journals, countries, and keywords were conducted using all available data under an inclusion threshold of 1. Collaboration networks between countries and keyword co-occurrence networks were constructed using VOSviewer (version 1.6.19; Centre for Science and Technology Studies, Leiden University) [9]. To enhance interpretability and network clarity, stricter thresholds were applied for network visualizations: keywords were retained only if they occurred at least 4 times, and country coauthorships were included only with a minimum of 5 occurrences. A keyword tree map was generated using the R package *Bibliometrix* (version 4.1.2) [10], with box sizes proportional to the keyword occurrence frequency. Keyword burst detection was conducted using CiteSpace (version 6.3.R1) [11] with the following parameters: g-index scale factor=2000, gamma=1, and minimum duration=1. The gamma parameter regulates the weight distribution in the burst strength calculation. A value of 1 ensures a balanced weighting scheme that neither overemphasizes recent citation spikes nor discounts significant earlier activity. The minimum duration parameter set to 1 requires that any identified citation burst persist for at least 1 year.

To evaluate journal impact, we considered both publication volume and citation metrics, and visualized the results using bubble plots. The x-axis represented log_10_(total publications+1), whereas the y-axis corresponded to log_10_(total citations+1). Bubble size reflected the average number of citations per publication, and color indicated the mean publication year, together providing a multidimensional perspective on journal influence.

To assess the influence of authors, we applied fractional counting to both publication and citation counts. For each author *A* who published *k* articles, we defined the following metrics:



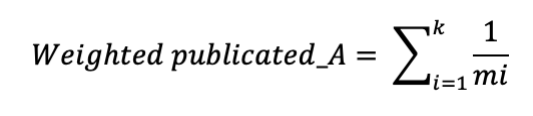



**(1)**



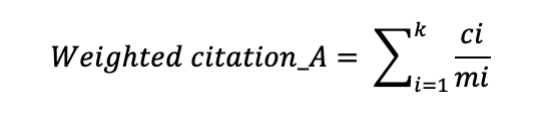



**(2)**

where *mi* denotes the number of authors for article *i* and *ci* represents the citation count for article *i*. This fractional counting approach prevents the inflation of metrics for authors who frequently publish in large collaborations, thereby ensuring a more equitable representation of individual scholarly contributions compared to whole-counting methods.

The cocitation clustering analysis was conducted using the R package *ComplexHeatmap* (version 2.12.1) [12]. A reference matrix was constructed, with rows representing cited references and columns representing citing documents. To focus on the most influential references, two inclusion criteria were applied: (1) cited references must be among the top 30 most frequently cited based on citation counts, and (2) citing documents must reference at least 2 of these top 30 cited references. This approach ensured the identification of meaningful cocitation patterns rather than isolated references. K-means clustering (*k*=3) was applied to the citing documents, with the algorithm repeated 500 times to ensure stability. The choice of 3 clusters was empirically determined to balance interpretability and granularity. Clusters were interpreted by examining cocitation patterns (which references frequently appeared together) and temporal trends (how citation networks evolved over time).

## Results

### Influential Documents, Journals, and Authors in the Field of AI in Medical Education

**Figure 2:**
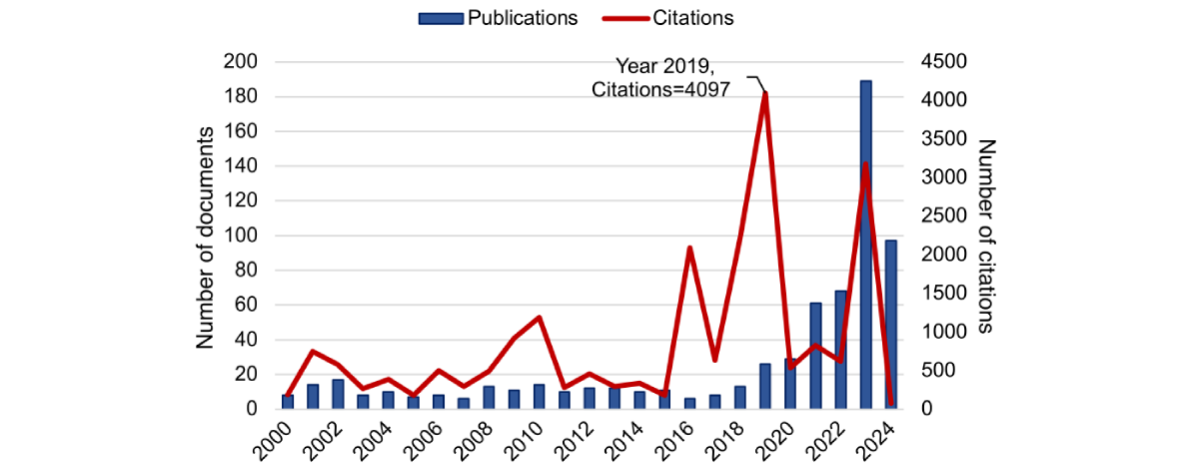
Trends in publication and citation numbers.

**Figure 3:**
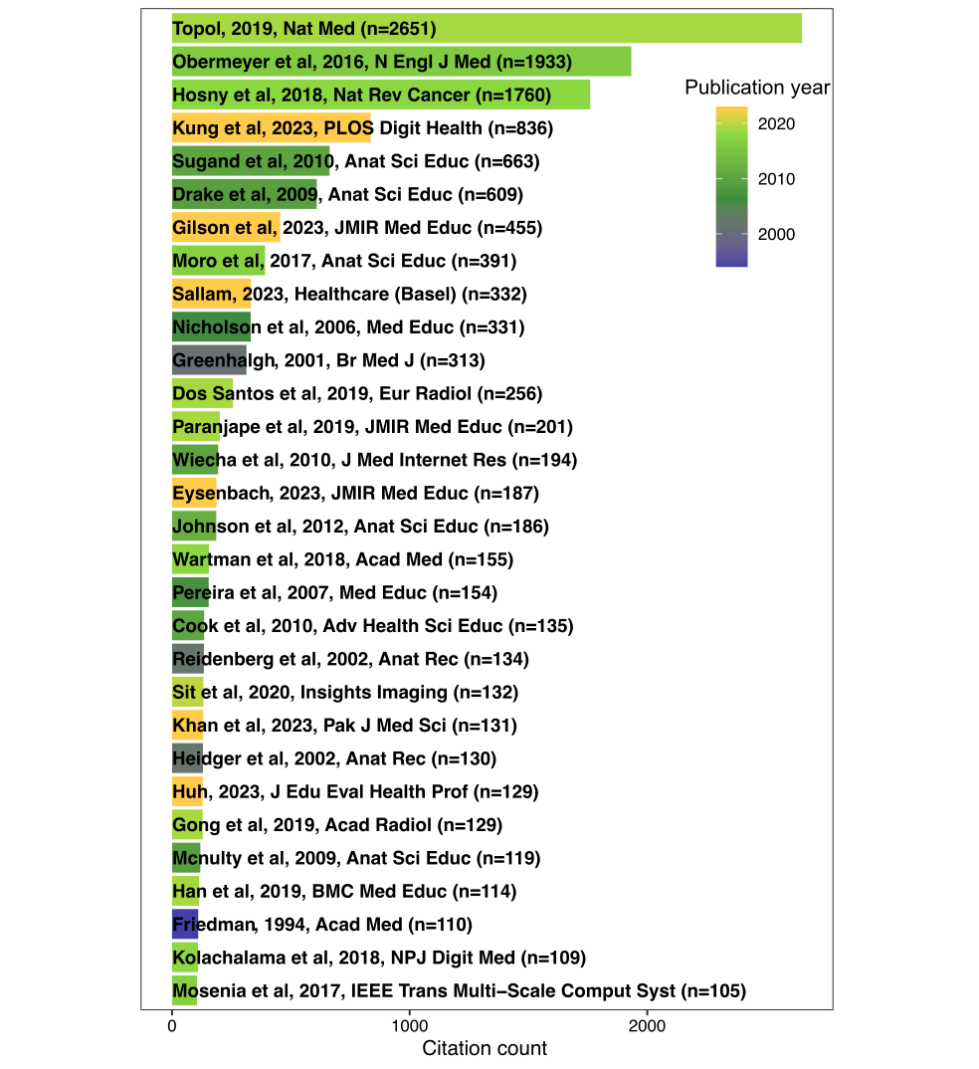
Top 30 most cited documents.

Our bibliometric analysis encompassed 669 documents from 269 journals authored by 3296 individuals across 295 countries, highlighting the global scope of research in this field. Figure 2 shows a notable increase in publications on AI in medical education since 2021. It is worth noting that, although publications from 2019 were relatively limited in number, they received substantial citations, suggesting that key conceptual foundations were proposed during this period. Figure 3 shows the top 30 most cited documents. Several highly influential works were published in 2019, most prominently the landmark article “High-Performance Medicine: The Convergence of Human and Artificial Intelligence” by Topol [13], which garnered 2651 citations. Other notable contributions included “Medical Students’ Attitude Towards Artificial Intelligence: A Multicentre Survey” by Pinto Dos Santos et al [14] (cited 256 times) and “Introducing Artificial Intelligence Training in Medical Education” by Paranjape et al [15] (cited 201 times), further underscoring that 2019 marked the emergence of critical discussions on AI integration. More recent highly cited works reflected growing interest in generative AI models, such as “Performance of ChatGPT on USMLE: Potential for AI-assisted medical education using large language models” by Kung et al [16] (cited 836 times) and “How Does ChatGPT Perform on the United States Medical Licensing Examination? The Implications of Large Language Models for Medical Education and Knowledge Assessment” by Gilson et al [17] (cited 455 times).

**Figure 4:**
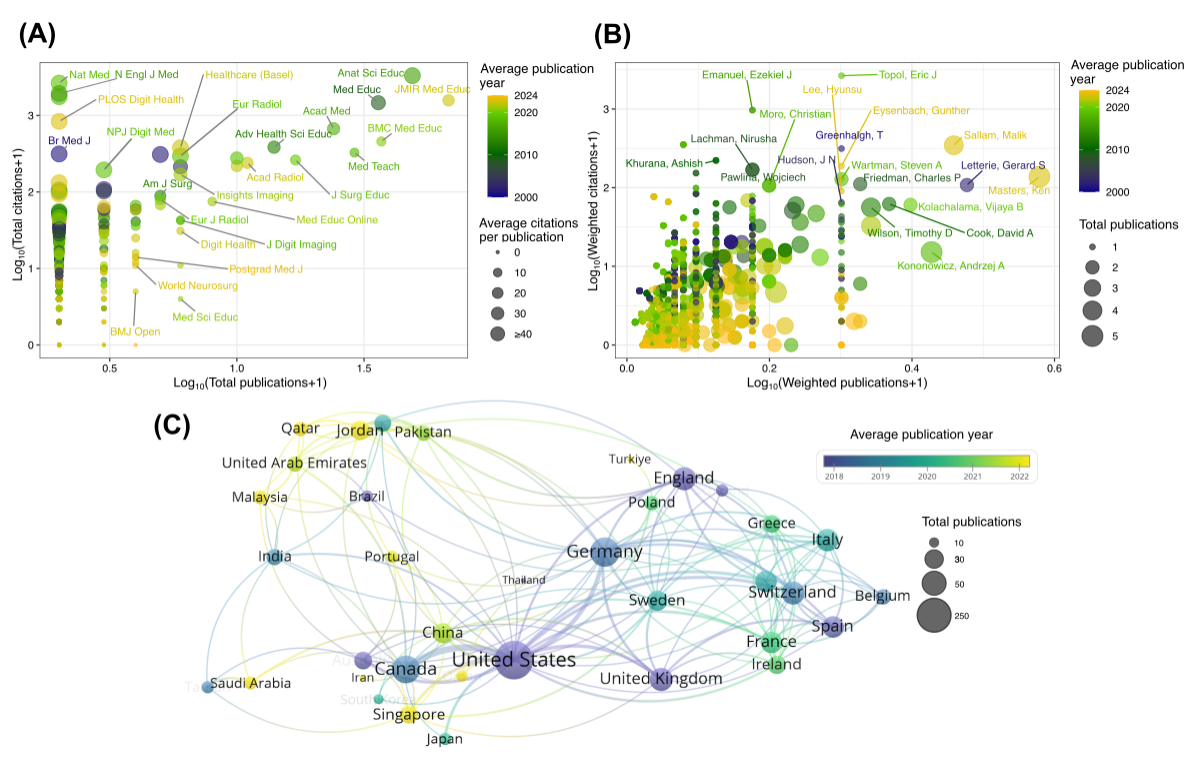
Journal, author, and country contributions visualized through bubble plots and a collaboration network. (A) For journals, bubble size reflects average citations per publication. (B) For authors, bubble size shows total publications. (C) In the country collaboration network, node size shows total publications, and line thickness represents partnership strength. Bubble and node colors reflect average publication year.

Next, we evaluated the contribution of journals, authors, and countries within the field of AI in medical education (Figure 4). Relying solely on citation metrics may overlook journals that make consistent and foundational contributions to the field. To address this limitation, we applied a log-transformation to both citation and publication counts, allowing for a more balanced and comparable assessment across journals. Our analysis identified the following as the most influential journals in AI in medical education: *JMIR Medical Education* (publications: 67/669, 10%; citations: n=1575), *Anatomical Sciences Education* (publications: 48/669, 7.2%; citations: n=3317), *Medical Education* (publications: 35/669, 5.2%; citations: n=1467), *BMC Medical Education* (publications: 36/669, 5.4%; citations: n=456), *Academic Medicine* (publications: 23/669, 3.4%; citations: n=669), and *Medical Teacher* (publications: 28/669, 4.2%; citations: n=326).

To assess the scholarly impact of authors while preventing the inflation of metrics for authors who frequently publish in large collaborations, we applied weighted counting to both publication and citation counts. Analogous to the normalization applied in journal-level analysis, we also used a log_10_ transformation to weighted citation and weighted publication counts at the author level. The results indicated that the following authors achieved high weighted publication and citation scores, reflecting their substantial contributions to the AI in medical education research field: Ken Masters (publications: 5/669, 0.7%; citations: n=140), Malik Sallam (publications: 4/669, 0.6%; citations: n=389), Gerard Letterie (publications: 2/669, 0.3%; citations: n=107), Steven Wartman (publications: 2/669, 0.3%; citations: n=254), Charles Friedman (publications: 2/669, 0.3%; citations: n=110), Kolachalama Vijaya (publications: 2/669, 0.3%; citations: n=114), Timothy Wilson (publications: 4/669, 0.6%; citations: n=176), David Cook (publications: 2/669, 0.3%; citations: n=151), and Andrzej Konowicz (publications: 5/669, 0.7%; citations: n=50).

To examine global collaboration patterns in the field, we constructed a country-level collaboration network, which revealed a central role for researchers based in the United States. The United States not only produced the highest volume of publications but also formed the most extensive collaborative ties with other countries. Similarly, several European nations—including Germany, the United Kingdom, and France—emerged as major contributors. By incorporating temporal information through the average publication year for each country, we observed that non-Western countries such as China, Qatar, and Malaysia are increasingly active in the field, reflecting its expanding global engagement.

### Identification of Research Hot Spots Using Keyword Analysis

**Figure 5:**
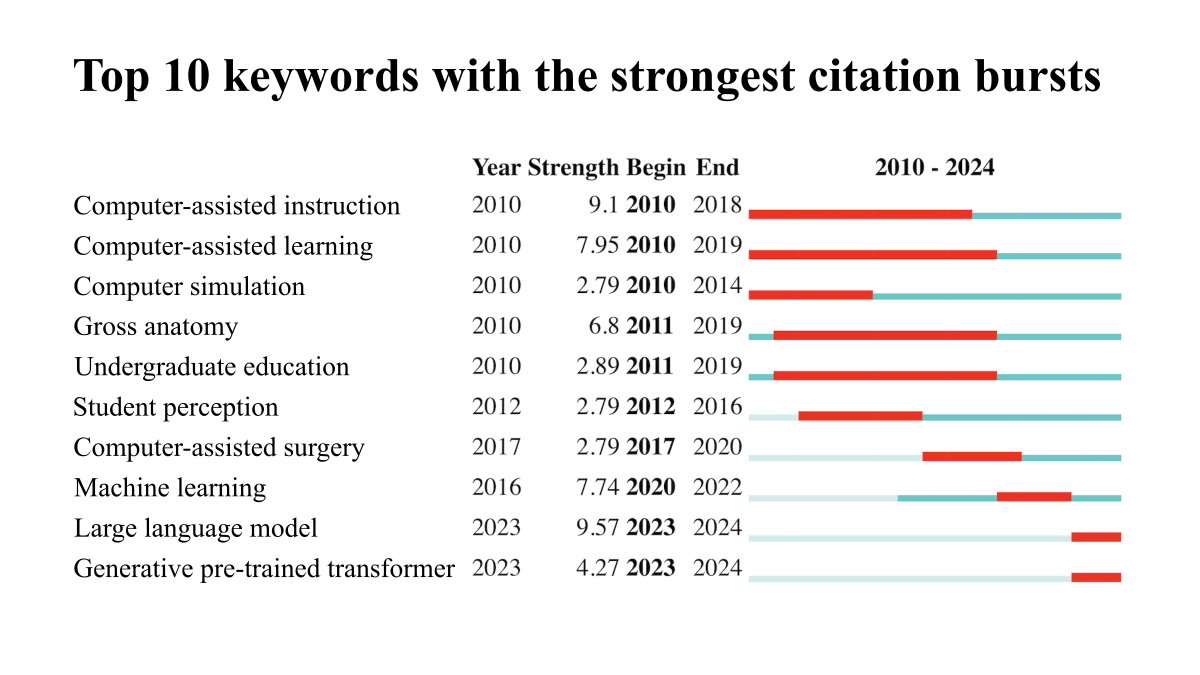
Keyword burst citation detection analysis indicating the year of emergence, burst strength, and the start and end years of each keyword’s citation surge.

To identify research hot spots over time, a keyword citation burst analysis was conducted. This method reveals when specific keywords become popular, how long they stay important, and the point at which their influence declines [18]. Figure 5 illustrates the top 10 keywords with the strongest citation bursts. Early bursts around 2010 featured terms such as “computer-assisted instruction” and “computer simulation,” largely within the context of gross anatomy courses, particularly in undergraduate education. Subsequent trends highlighted the expansion of AI-enabled techniques into computer-assisted surgery. In 2023, research attention shifted toward “machine learning” and “large language models,” with the generative pretrained transformer model standing out as one of the most promising developments likely to shape the future of the field.

**Figure 6:**
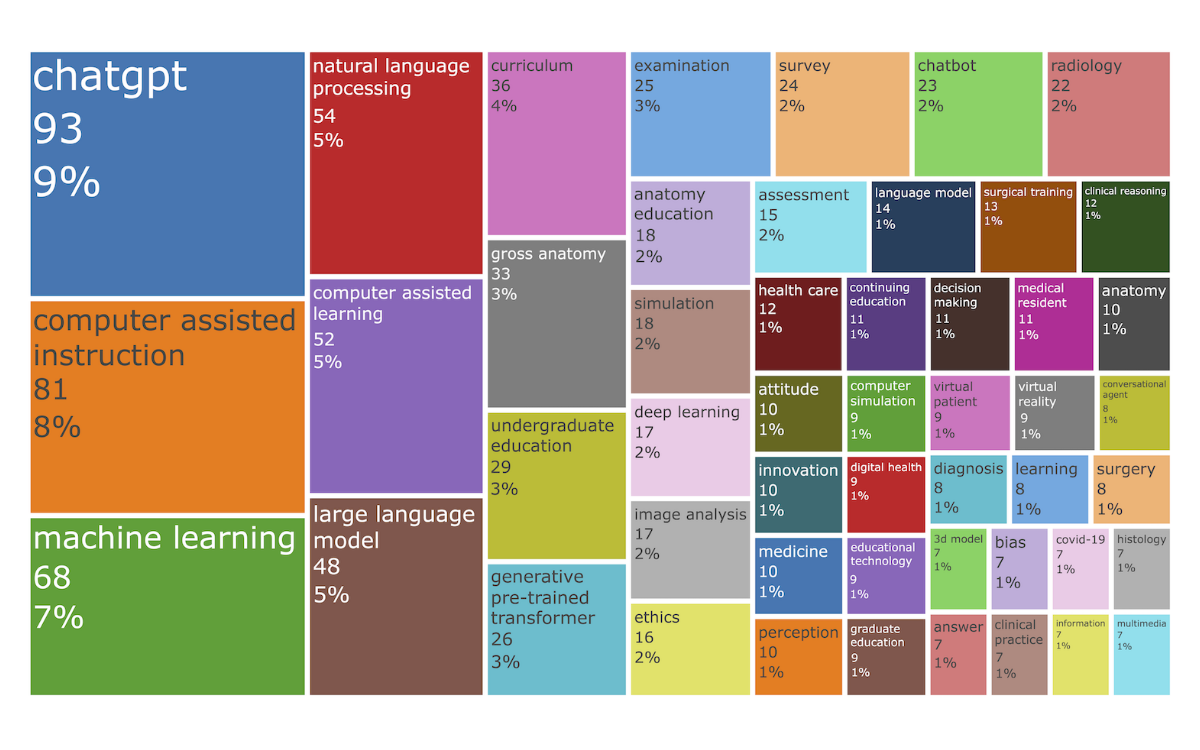
Tree map of author keywords sized by their frequency of occurrence in the literature.

A keyword tree map was constructed to visualize the frequency of keywords across the collected documents (Figure 6). The most common keywords included “ChatGPT,” “computer-assisted instruction,” and “machine learning.” Frequent applications for AI in medical education were associated with anatomy, curriculum design, simulation, and image analysis. In addition, topics such as ethics, health care, and digital health also emerged as focal areas within AI-based educational research.

**Figure 7:**
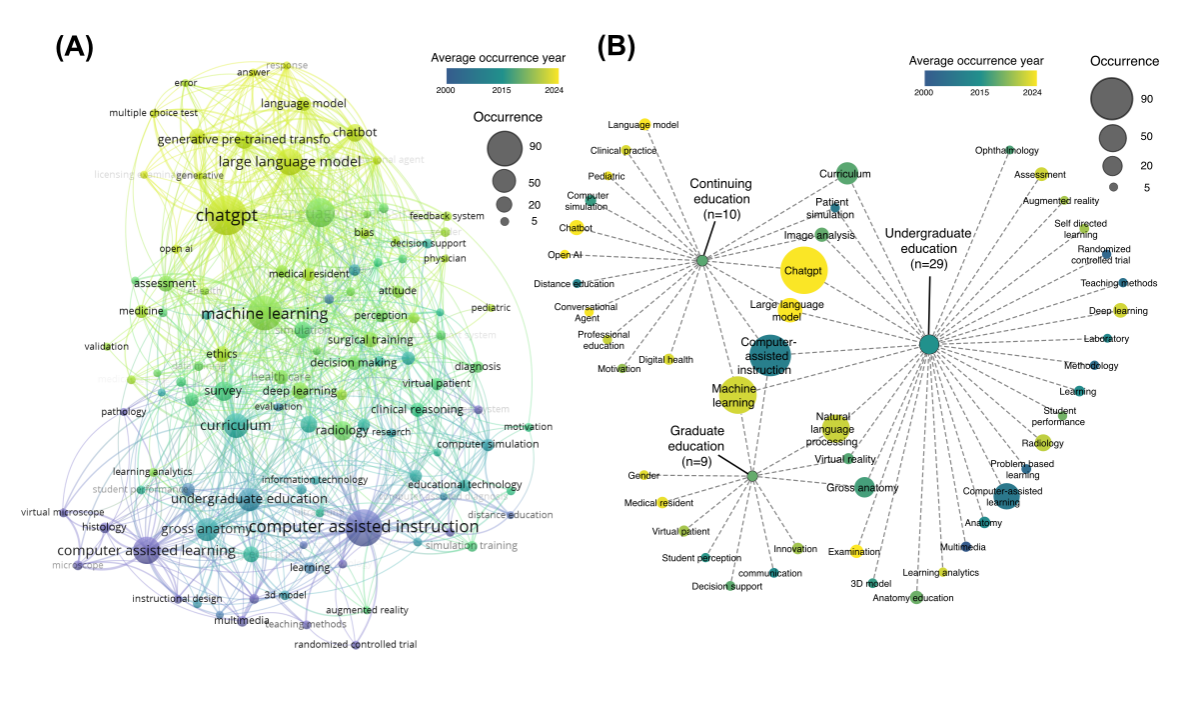
Keyword co-occurrence network. (A) Node size corresponds to keyword frequency, and node color represents the average occurrence year (blue represents older terms; yellow indicates more recent terms). (B) Magnified view of the network highlighting keywords associated with undergraduate, graduate, and continuing medical education.

To gain deeper insights into conceptual relationships and their evolution, we developed keyword co-occurrence networks. Temporal evolution of the co-occurrence network aligned with the burst citation analysis (Figure 7). Computer-assisted learning was linked to various teaching techniques, including virtual microscopy, patient simulation, and 3D models. More recently, with the development of machine learning approaches, research interest shifted toward wider applications, such as curriculum reform, big data analysis, health care practice, and surgical training. The network further highlights natural language processing and large language models as current research hot spots, particularly in applications such as answering multiple-choice tests in medical licensing examinations, providing feedback systems, and serving as conversational agents. We then compared co-occurrence patterns across undergraduate education (occurrence: n=29; average occurrence year 2013, SD 7.8), graduate education (occurrence: n=9; average occurrence year 2016, SD 8.5), and continuing medical education (occurrence: n=10; average occurrence year 2016, SD 7.2). The results revealed that anatomy education, examinations, and problem-based learning were prominent in undergraduate medical education; graduate education emphasized communication, innovation, and decision support; and continuing medical education focused on clinical practice and digital health. Notably, large language models such as ChatGPT permeated all 3 educational stages.

### Cocitation Analysis of References

**Figure 8:**
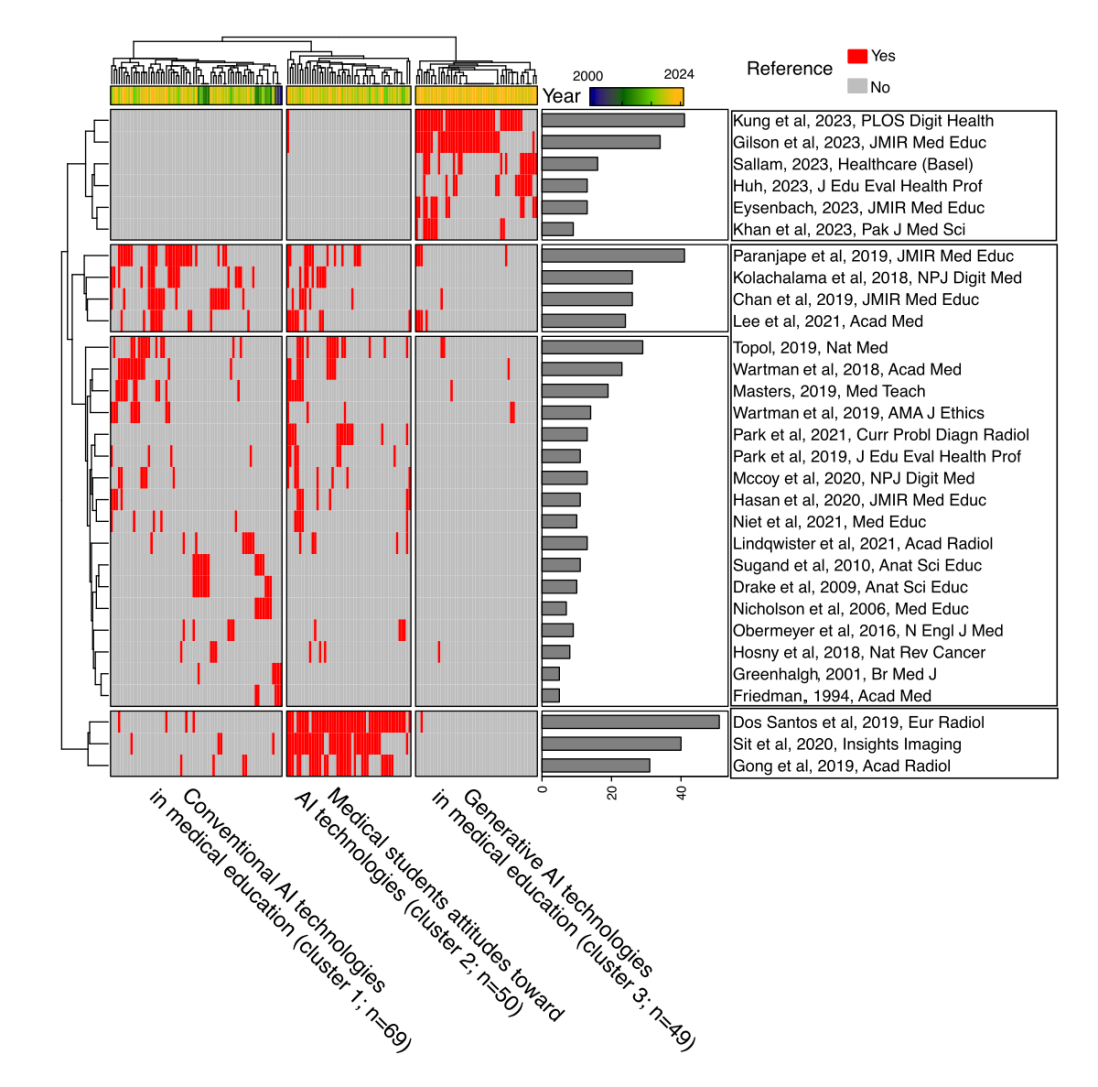
The cocitation structure of the literature. Rows represent cited references, whereas columns denote citing articles. Red cells indicate the presence of a cocitation relationship, and the temporal gradient (blue represents older terms; yellow indicates more recent terms) highlights the chronological evolution of citation patterns. The accompanying bar plot reflects the cumulative citation frequency of each reference (Multimedia Appendix 3). AI: artificial intelligence.

Next, we examined the cocitation structure among the documents. Studies with shared research interest tended to cite similar foundational references, revealing their intellectual lineages [11]. A reference co-occurrence matrix was constructed, where rows represented cited references and columns represented citing articles. To capture meaningful cocitation patterns rather than isolated references, we included only the top 30 most frequently cited references and required that citing documents reference at least 2 of these highly cited works. After applying these criteria, 25.1% (168/669) of the articles remained for subsequent analysis. This approach identified 3 principal thematic clusters within the literature (Figure 8). The first was conventional AI technologies in medical education (cluster 1; citing documents: 69/168, 41.1%). References in this cluster advocated for structural innovations in medical training, with an emphasis on integrating computational thinking and AI literacy (Paranjape et al [15], Chan and Zary [2], Kolachalama and Garg [19], Topol [13], Wartman and Combs [20,21], and Masters [22]). The second thematic cluster was medical students’ attitudes toward AI technologies (cluster 2; citing documents: 50/168, 29.8%). Studies in this cluster used quantitative surveys and qualitative methods to evaluate learner engagement with emerging tools (Pinto Dos Santos et al [14], Gong et al [23], and Sit et al [3]). The third thematic cluster was generative AI technologies in medical education (cluster 3; citing documents: 49/168, 29.2%). A rapidly growing cluster, it addressed implementation challenges and validation frameworks for generative AI models (Sallam [1], Huh [24], Kung et al [16], and Gilson et al [17]). Notably, while recent publications showed substantial emphasis on generative AI applications (cluster 3), few studies comparatively evaluated generative AI technologies against earlier conventional AI education reforms (cluster 1) or prioritized investigating student attitudes (cluster 2) toward these new tools.

## Discussion

### Emerging Trends and Global Contributions in AI-Driven Medical Education Research

The analysis of influential works in AI-based medical education revealed that 2019 was a critical turning point for the field. Seminal publications by Topol [13], Pinto Dos Santos et al [14], and Paranjape et al [15] laid the conceptual foundation for AI integration into medical training, sparking sustained scholarly dialogue. Their high citation rates reflect broad recognition of issues such as digital competency development, curricular reform, and learner attitudes toward AI—themes that remain relevant today. The more recent surge in citations for works on large language models (eg, Kung et al [16] and Gilson et al [17]) highlights a shift toward generative AI applications, particularly in knowledge assessment and adaptive learning platforms. This pattern aligns with global trends in AI adoption, where emerging technologies rapidly influence educational paradigms.

Our journal-level analysis further demonstrates the importance of using normalized metrics to evaluate influence. While journals such as *Nature Medicine* and the *New England Journal of Medicine* achieved high citation counts per article—often due to overarching prestige or coverage of breakthrough topics—specialized journals such as *JMIR Medical Education*, *Anatomical Sciences Education*, and *Medical Education* provided consistent intellectual contributions that shaped the field structurally. This result is consistent with previous literature findings that *JMIR Medical Education* and *Medical Teacher*, among others, are ranked as the top most productive journals [25]. The log-transformation approach helped mitigate biases toward low-output, high-impact journals, offering a more equitable assessment of sustained influence.

At the author level, weighted counting methods revealed key contributors without overrepresenting researchers involved predominantly in large collaborations. Scholars such as Ken Masters, Malik Sallam, and Steven Wartman produced focused yet high-impact work, suggesting that quality and thematic consistency can achieve significant scholarly recognition even with modest publication numbers.

Geographically, the United States and Western European nations currently lead in output and collaboration reach. However, the rising activity from countries such as China, Qatar, and Malaysia signals a broadening of research capacity and interest beyond traditional hubs. This decentralization may foster diverse perspectives and context-specific AI applications in global medical education.

### Evolution of Thematic Focus and Implications of AI in Medical Education

The integration of AI into medical education has undergone distinct thematic shifts over the past 2 decades, reflecting both technological advancements and evolving pedagogical priorities. In the early 2000s, efforts centered on computer-assisted instruction, leveraging multimedia, 3D models, and virtual reality to enhance anatomy instruction and patient simulation [26,27]. These tools initially focused on cognitive skill development but often overlooked social and psychomotor competencies essential to clinical practice. Our keyword analysis validated this trajectory, showing early citation bursts around 2010 for terms such as “computer-assisted instruction” and “computer simulation,” particularly within undergraduate anatomy education. Subsequent developments saw AI applications expand into computer-assisted surgery and more integrated educational supports, including systems for personalized learning and clinical reasoning simulation [15,19]. Our co-occurrence network analysis further delineated these thematic alignments across different training stages. The undergraduate education phase, with an average keyword occurrence year of 2013, emphasized foundational topics such as anatomy, examinations, and problem-based learning. In contrast, graduate education, with an average keyword occurrence year of around 2016, reflected a shift toward communication skills and clinical decision support. Meanwhile, continuing medical education, also centered around 2016, focused strongly on clinical practice and digital health. Together, these patterns illustrate AI’s expanding role in supporting the entire continuum of medical training.

A significant shift occurred around 2023, as observed in both our burst detection and co-occurrence networks, with research attention toward “machine learning” and “large language models.” Keywords such as “ChatGPT” and “generative AI” now dominate the literature, reflecting a new wave of innovation aimed at conversational agents, feedback systems, and assessment tools, particularly in answering multiple-choice questions and simulating tutor-learner interactions. Notably, large language models have permeated all levels of medical education, suggesting their transformative potential across undergraduate, graduate, and continuing training contexts [17,28].

### Challenges in AI-Driven Medical Education and Future Directions

AI can automate routine tasks, alleviating the cognitive load on learners’ working memory. This process of “cognitive off-loading” enables students to dedicate more mental resources to mastering higher-order, complex skills [29]. Consequently, modern medical curricula should transition from rote memorization to emphasizing higher-order skills such as computational thinking and AI literacy [21]. However, keyword analysis indicates that current AI in medical education remains predominantly focused at the undergraduate level, with keyword frequencies of 29 for undergraduate education compared to only 9 for graduate education and 10 for continuing medical education. This disparity suggests significant potential for expanding AI integration into both graduate and continuing medical education [30]. Proposed reforms include providing foundational training in statistics, offering hands-on experience with open-source tools, and integrating AI components into existing courses [2,31]. A structured, stage-specific educational approach is recommended across all training levels: premedical education should introduce AI concepts in entrance examinations, medical training should require coursework in data science and AI ethics, clinical rotations should incorporate AI decision support tools (eg, radiology AI), and postgraduate training should include continuing education modules on emerging AI applications [13,15]. Nevertheless, overreliance on AI carries the risk of eroding fundamental medical skills among students [29]. A balanced approach is essential to ensure that AI serves as a complement to rather than a replacement for core clinical competencies.

Cocitation analysis revealed that cluster 1 corresponded to curriculum reforms and recommendations proposed by medical education experts for structural innovations in medical training [2,13,15,19-22], whereas cluster 2 comprised studies exploring students’ attitudes toward AI applications in medical education [3,14,23]. However, there is a noticeable gap between recent research on generative AI (cluster 3) and the earlier AI in medical education studies (clusters 1 and 2), indicating that integrative frameworks incorporating learning theories and user feedback remain underdeveloped. Future research should prioritize the development of unified theoretical models that incorporate diverse learning perspectives into generative AI systems, as well as establish assessment frameworks to evaluate the effectiveness of generative AI in achieving intended educational outcomes. Moreover, the perceptions and attitudes of both teachers and students are critical for technological advancement, underscoring the importance of a co-design approach in developing educational tools [20]. Therefore, it is essential to evaluate the efficacy of AI integration into existing curricula using multidimensional metrics, including surveys and learning analytics [14,32].

We also observed that the keyword “ethics” (occurrence: n=16) represented a significant theme in AI in medical education, frequently co-occurring with terms such as “big data,” “bias,” and “privacy.” Data privacy and model reliability remained major concerns, especially when processing a large volume of sensitive information [2]. Furthermore, skepticism persists due to issues such as potential algorithmic bias and AI’s limited adaptability to diverse cultural and contextual settings within medical education [33]. To address these challenges, AI systems should emphasize explainability and incorporate human oversight, enabling educators to review and override unjustified decisions [34]. Robust data governance and ethical guidelines must form the foundation of AI deployment, whereas interdisciplinary collaboration helps ensure that AI solutions are aligned with educational goals, whereas interdisciplinary collaboration helps ensure that AI solutions are aligned with educational goals [19]. Involving both instructors and students in the development and validation of AI tools can lead to more adaptive and context-aware systems that effectively complement human expertise and deliver targeted, AI-enhanced educational support.

### Limitations

Potential methodological limitations should be considered, including language bias, the exclusion of gray literature, and the artificial temporal boundary set for the analysis (2000-2024). Due to the inherent constraints of bibliometric methods, our findings should be interpreted as exploratory in nature. It is also important to note that the identified trends and research hot spots are derived from publication keywords. To address these limitations, future studies should use a more comprehensive content analysis framework. This approach will facilitate a deeper exploration of broader trends in AI applications, such as their objectives, methodologies, and major outcomes, thereby yielding richer insights than those of traditional bibliometric techniques.

### Comparison With Prior Work

Previous bibliometric studies have explored AI applications in medical education [6,25]. Many of their findings can be reproduced, such as author impact, identification of influential journals, international collaboration patterns, and keyword frequency analyses. However, our study introduced several novel methodological approaches. First, by using the search strategy proposed by Maggio et al [7], we identified a broader corpus of literature in the domain of AI in medical education. Second, we investigated the temporal shift in the average publication year across journals, authors, and countries, as well as the evolution in research themes. This approach allowed for dynamic tracking of knowledge contributions and thematic trends over time. Third, while Wang et al [6] summarized the top 10 most cited references in their dataset, a list that shows considerable overlap with our reference matrix, we extended this analysis by visualizing the cocitation network structure using a clustered heat map. This revealed distinct clustering among citing articles, offering deeper structural insights into the intellectual foundations of the field. Our cocitation methodology is designed to be generalizable and applicable to other bibliometric research domains.

### Conclusions

As AI technologies continue to evolve, they are likely to enable more sophisticated simulations, personalized learning experiences, and data-driven insights that may transform medical education. Our bibliometric analysis provides an exploratory overview of current research trends and collaborations in this emerging domain, offering preliminary insights that can guide future, more in-depth investigations. Future research in AI-based medical education should prioritize expanding into graduate and continuing medical training, bridging the disciplinary gap between generative AI and established medical AI research through integrated frameworks, and deepening inquiry into learner and educator perceptions to ensure the responsible and effective implementation of these technologies.

## Appendix

Multimedia Appendix 1 Medical Education Journal List 24.

Multimedia Appendix 2 Detailed search strings for data extraction.

Multimedia Appendix 3 Dataset of the summarized 669 documents included in the analysis.

Multimedia Appendix 4 Mapping of keyword transformations.

## Abbreviations

AI: artificial intelligence
DOI: digital object identifier
MEJ-24: Medical Education Journal List 24

## Data Availability

All data generated or analyzed during this study are included in this published article and its supplementary information files (Multimedia Appendices 1 to 4).
